# Expression of 5-HT_3_ receptors and TTX resistant sodium channels (Na_V_1.8) on muscle nerve fibers in pain-free humans and patients with chronic myofascial temporomandibular disorders

**DOI:** 10.1186/1129-2377-15-63

**Published:** 2014-09-26

**Authors:** Nikolaos Christidis, Isabell Kang, Brian E Cairns, Ujendra Kumar, Xudong Dong, Annika Rosén, Sigvard Kopp, Malin Ernberg

**Affiliations:** 1 Orofacial Pain and Jaw Function, Department of Dental Medicine, Karolinska Institutet, Huddinge, Sweden; 2 Scandinavian Center for Orofacial Neuroscience (SCON), Stockholm, Sweden; 3 Faculty of Pharmaceutical Sciences, University of British Columbia, Vancouver, Canada; 4 Center for Sensory-Motor Interaction, Aalborg University, Aalborg, Denmark; 5 College of Stomatology, Tianjin Medical University, Tianjin 300070, P. R. China; 6 Oral and Maxillofacial Surgery, Department of Dental Medicine, Karolinska Institutet, Huddinge, Sweden; 7 Oral and Maxillofacial Surgery, Department of Clinical Dentistry, University in Bergen, Bergen, Norway

**Keywords:** Muscle biopsy, Human subjects, Myofascial TMD, 5-HT_3_-receptor, Na_V_1.8 sodium channel

## Abstract

**Background:**

Previous studies have shown that 5-HT_3_-antagonists reduce muscle pain, but there are no studies that have investigated the expression of 5-HT_3_-receptors in human muscles. Also, tetrodotoxin resistant voltage gated sodium-channels (Na_V_) are involved in peripheral sensitization and found in trigeminal ganglion neurons innervating the rat masseter muscle. This study aimed to investigate the frequency of nerve fibers that express 5-HT_3A_-receptors alone and in combination with Na_V_1.8 sodium-channels in human muscles and to compare it between healthy pain-free men and women, the pain-free masseter and tibialis anterior muscles, and patients with myofascial temporomandibular disorders (TMD) and pain-free controls.

**Methods:**

Three microbiopsies were obtained from the most bulky part of the tibialis and masseter muscles of seven and six healthy men and seven and six age-matched healthy women, respectively, while traditional open biopsies were obtained from the most painful spot of the masseter of five female patients and from a similar region of the masseter muscle of five healthy, age-matched women. The biopsies were processed by routine immunohistochemical methods. The biopsy sections were incubated with monoclonal antibodies against the specific axonal marker PGP 9.5, and polyclonal antibodies against the 5-HT_3A_-receptors and Na_V_1.8 sodium-channels.

**Results:**

A similar percentage of nerve fibers in the healthy masseter (85.2%) and tibialis (88.7%) muscles expressed 5-HT_3A_-receptors. The expression of Na_V_1.8 by 5-HT_3A_ positive nerve fibers associated with connective tissue was significantly higher than nerve fibers associated with myocytes (*P* < .001). In the patients, significantly more fibers per section were found with an average of 3.8 ± 3 fibers per section in the masseter muscle compared to 2.7 ± 0.2 in the healthy controls (*P* = .024). Further, the frequency of nerve fibers that co-expressed Na_V_1.8 and 5-HT_3A_ receptors was significantly higher in patients (42.6%) compared to healthy controls (12.0%) (*P* < .001).

**Conclusions:**

This study showed that the 5-HT_3A_-receptor is highly expressed in human masseter and tibialis muscles and that there are more nerve fibers that express 5-HT_3A_-receptors in the masseter of women with myofascial TMD compared to healthy women. These findings indicate that 5-HT_3_-receptors might be up-regulated in myofascial TMD and could serve as potential biomarkers of chronic muscle pain.

## Background

Serotonin (5-HT) is an important biogenic amine that fulfills the role of a neurotransmitter and neuromodulator, and is an important mediator of pain both peripherally and centrally [1,2]. Several studies have shown that 5-HT concentrations are significantly elevated in painful muscles of patients with chronic myalgia [3-6]. In the periphery, 5-HT has been shown to excite and sensitize nociceptors, and in humans to cause muscle pain and sensitivity [7]. Excitation of nociceptors by 5-HT may be due to either a direct effect, such as activation of the 5-HT_3_ receptor, or an indirect effect, which may involve the release of other algogens, such as substance P or glutamate [8,9]. In humans, evidence suggests that 5-HT activates the 5-HT_3_-receptor to mediate muscle pain and sensitivity to mechanical stimulation [7,10-14].

Five subunits of the 5-HT_3_-receptor have been identified (5-HT_3A_ – 5-HT_3E_). The 5-HT_3A_ subunit forms a complete receptor, while the other subunits can only form functional receptor complexes together with the 5-HT_3A_ subunit [15-17]. Results from some studies indicate that intramuscular injections of 5-HT_3_ antagonists may be effective for treatment of various muscular pain conditions [18-20]. The 5-HT_3_ antagonist granisetron has been shown to reduce pain and allodynia induced by 5-HT or hypertonic saline [10,13] and also to increase the pressure pain threshold (PPT) over healthy muscles in human experimental studies [10,11,21]. In a recent study, we reported that approximately 45-50% of the muscle nerve fibers of both healthy humans and rat expressed the NMDA receptor subunit 2B [22] but the expression of 5-HT_3_-receptors in human muscles has not yet been investigated nor if the frequency is altered in painful muscles. Hence, it is not clear if the pain-reducing effect of these 5-HT_3_ antagonists is only due to blocking of 5-HT_3_ receptors or if they act on other receptors as well.

The tetrodotoxin (TTX) resistant sodium channels (Na_V_s) have been shown to be highly expressed in small neurons of the dorsal root ganglia (DRG) in both human and animal studies considered to be probable nociceptors [23]. The Na_V_1.8 channel has also been found in the soma of small diameter sensory C and Aδ neurons [24] which have been shown to be involved in nociception in models of neuropathic and inflammatory pain [25]. One study reported that the Na_V_1.8 channel is present in 86% of the muscle sensory afferent fibers, which makes it a useful marker of putative muscle nociceptors [26], and recently it has been found in trigeminal ganglion neurons innervating the rat masseter muscle [27]. Taken together, this evidence suggests that Na_V_1.8 channel mainly found in the peripheral sensory neurons and their expression on nerve fibers provides a method to identify putatively nociceptive afferent fibers [24,28]. The Na_V_1.8 has been shown to participate in nociception and peripheral sensitization, and therefore is thought to be an important for chronic pain [23-25,29,30]. It has further been shown that inflammatory mediators, such as serotonin, increase the excitability of sensory neurons by modulating the Na_V_1.8 [31-33]. Therefore, Na_V_1.8 is particularly interesting both as a marker of putative nociceptive afferent fibers and as an additional target/mechanism to explain the analgesic effects of local administration of the 5-HT_3_ receptor antagonists [10,18-21].

The purpose of this study was to investigate the expression of 5-HT_3A_-receptors and Na_V_1.8 sodium channels in nerve fibers of human masseter and tibialis muscles, since results from both animal and human experimental studies suggest that 5-HT_3_ receptor antagonists reduce musculoskeletal pain and hyperalgesia [18-20]. Further, it is not known if the 5-HT_3_ receptor is expressed in painful muscle tissues of humans. If so, it could offer an important target for novel treatment approaches for these patients and provide important information about the pathophysiology of muscle pain especially in the temporomandibular region. Therefore, the study also aimed to investigate the frequency of nerve fibers that co-express the 5-HT_3A_-receptors and Na_V_1.8 sodium channels and to compare the frequency 1) between healthy pain-free men and women; 2) between patients with myofascial temporomandibular disorders (TMD) and pain-free, healthy, sex- and age-matched controls; and 3) between the masseter and tibialis anterior muscles (a muscle seldom affected by chronic pain) of healthy men and women. An additional aim of the study was to investigate if a minimal invasive microbiopsy technique is equivalent to the traditional open biopsy technique for immunohistochemical analyses of receptor expression in human muscles.

## Methods

The methods and selection of participants were approved by the regional ethical review board in Stockholm, Sweden, (2007/2:6). All participants were above 20 years of age and the study followed the principles for medical research according to the guidelines of the Declaration of Helsinki as well as the Good Clinical Practice guidelines.

At a separate visit the participants were screened for trial suitability with a clinical examination performed in accordance with the Research Diagnostic Criteria for TMD (RDC/TMD [34]) after information about the study protocol. The participants received both written as well as verbal information and gave their verbal and written consent.

### Participants

Twenty three healthy volunteers and five patients with myofascial TMD were included in the study. The patients were recruited from patients referred to the section for Orofacial Pain and Jaw function, Department of Dental Medicine, Karolinska Institutet, Huddinge, Sweden, while the healthy volunteers were recruited from colleagues at the Department of Dental Medicine. The biopsy procedure took place at the Department of Dental Medicine, Karolinska Institutet, Huddinge, Sweden at the section for Orofacial Pain and Jaw Function as well as at the section of Oral and Maxillofacial Surgery.

The distribution of the participants, their age and the duration of the local pain are shown in Table 1.

**Table 1:** The distribution of the age-matched participants divided by biopsy technique and sex

	**Microbiopsy**		**Traditional open biopsy**	
	**Healthy -*Masseter***	**Healthy - *Tibialis***	**Healthy**	**Patients**
**Number of participants**				
All	12	14	5	5
Men	6	7	0	0
Women	6	7	5	5
**Age**				
All	34.6 (±10.1)	37.4 (±11.8)	52.0 (±8.5)	50.4 (±8.2)
Men	34.8 (±9.9)	37.7 (±11.9)	-	-
Women	34.3 (±11.2)	37.1 (±12.6)	52.0 (±8.5)	50.4 (±8.2)
**Duration of pain**				
Men				-
Women				3.0 (±2.6)
**Intensity of pain**				
Men				-
Women				5.0 (±1.25)
**Co-morbid headache**				
Men				-
Women				5

The table displays the number (n) of participants, their mean (±SD) age (years), median (IQR) duration of pain (years), current median (IQR) intensity of pain (NRS), as well as number (n) of patients with co-morbid headache. The 12 participants in the microbiopsy masseter group are the same individuals as the 12 out of the 14 in the tibialis group.

NRS = Numeric rating scale (0–10).

IQR = Interquartile range (75 percentile minus 25 percentile).

### Inclusion criteria

The inclusion criteria for the healthy volunteers were age over 20 years and good general health.

The inclusion criteria for the patients were age over 20 years, a diagnosis of myofascial pain according to RDC/TMD criteria and pain, upon digital palpation, of at least one of the masseter muscles.

### Exclusion criteria

The exclusion criteria for both the healthy volunteers and the patients were diagnosis of: **a)** systemic muscular or joint diseases (e.g. fibromyalgia, rheumatoid arthritis, ankylosing spondylitis, psoriatic arthritis); **b)** whiplash associated disorder; **c)** neuropathic pain or neurological disorders (e.g. myasthenia gravis, craniomandibular dystonia), or report of: **d)** pain of dental origin; **e)** pregnancy; **f)** frequent use of muscle relaxants; and **g)** use of analgesic or anti-inflammatory medication during the 24 hours preceding biopsy.

An additional exclusion criterion for the healthy volunteers was facial pain or palpatory tenderness of the masseter or tibialis muscles.

### Biopsy-procedures

#### *Microbiopsies*

Microbiopsies were obtained from the masseter and tibialis anterior muscles from seven healthy men and seven age-matched healthy women. During the analysis process two biopsies from the masseter muscle were misplaced (from one man and one woman). The microbiopsies were taken through the skin overlaying the most prominent part of the masseter muscle and the tibialis anterior muscle. This procedure was performed under skin surface anesthesia using a prefabricated anesthetic patch (EMLA Patch®, 25 mg lidocaine and 25 mg prilocaine, AstraZeneca, Södertälje, Sweden) that was placed over the area to be penetrated, for a duration of 30 min. A disposable Monopty®Bard® biopsy instrument with a penetration depth of 11 mm and a diameter of 18G was used for the masseter muscle, while for the tibialis anterior muscle a diameter of 16G was used. The biopsy instrument was guided with a Bard®TruGuide™ coaxial needle (BARD Norden, Helsingborg, Sweden), that was inserted to a depth of 10 mm. This biopsy system is automated and of a type that has been proven to be effective i.e. for the diagnosis of musculoskeletal sarcomas [35].

Three microbiopsies were taken from each muscle in order to ensure that sufficient muscle tissue was obtained. The coaxial needle was inserted along the near long axis of the muscles until the fascia was penetrated and the biopsy instrument was then inserted through the coaxial needle. By pressing the trigger button the piston of the needle collected a piece of the muscle with a size of approximately 0.12 cm * 1.1 cm in the masseter and 0.14 cm * 1.1 cm in the tibialis anterior. The biopsy instrument was then slid out from the coaxial needle while the latter was maintained in place, thus avoiding repeated skin punctures. The muscle section was removed from the biopsy instrument using a sterile probe, and the biopsy instrument was rinsed with isotonic saline. This procedure was repeated twice. Each time the biopsy instrument was rotated 45° so that the microbiopsy would be taken from a new portion of the muscle.

#### *Traditional open biopsies*

Traditional open biopsies were obtained intra-orally from the masseter muscle of five myofascial TMD patients (all women) and from five healthy, age-matched women (not participating in the microbiopsy part). In the patients the most painful spot of the masseter muscle was aimed at, while in the healthy volunteers the spot selected coincided with the most painful spot in the patient that they were to be compared with.

After anesthesia with 1 mL lidocaine-adrenalin (20 mg/mL + 12.5 μg/mL) the oral mucosa was incised with a scalpel to expose the masseter muscle. The selected section was then cut out and removed from the muscle with a forceps. Two vicryl-sutures were placed over the incision and removed after one week.

The biopsies from the healthy volunteers were also used to validate the minimal invasive microbiopsy technique. This was done by investigating if the muscle sections obtained with the two different techniques were equivalent regarding the weight and size of the obtained biopsies, the amount of surrounding tissue in the biopsies, receptor expression, the subjective post-surgical discomfort and the participants’ experience of the procedures as well as the area of the wound.

### Analysis of the biopsies

#### *Blinding of samples*

The microbiopsies were obtained and coded by NC (DDS, PhD, specialist in orofacial pain and jaw function), the human traditional open biopsies were obtained by AR (DDS, PhD, Professor, specialist in oral and maxillofacial surgery) and coded by NC. The codes were not revealed to the person (IK) analyzing the samples.

#### *Immunohistochemistry*

**Figure 1:**
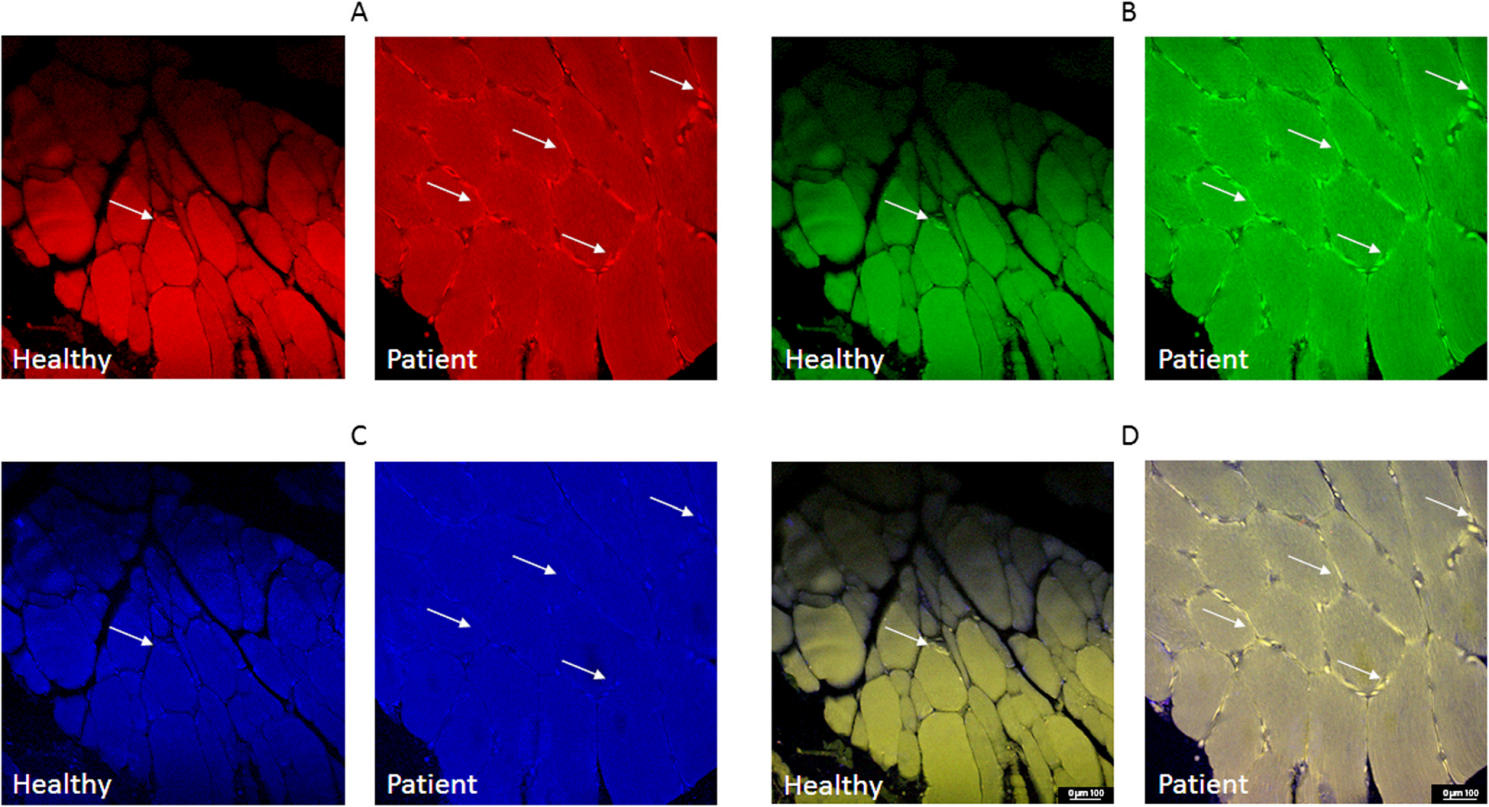
**Example of high power (40 x) photomicrographs for a masseter section from one female patient with myofascial TMD and a healthy age- and sex-matched pain-free volunteer.** The fibers shown are positively labelled for PGP 9.5 **(A)**, 5-HT_3A_**(B)**, and Na_V_1.8 **(C). (D)** is the composite image. Calibration bar: 100 μm.

Both the microbiopsies and the open biopsies were fixed with 4% paraformaldehyde at 4°C over-night, rinsed in phosphate-buffered saline (PBS), and then dehydrated, embedded in paraffin and sectioned at a thickness of 10 μm. The sections were mounted on glass slides, stored at 37°C over-night, dipped into xylene to remove the paraffin and rehydrated by being rinsed in ethanol (100%, 90% and 70%). The sections were then boiled in 10 mM (pH 6.0) citrate buffer for 10 minutes and rinsed as well as stored in PBS. Subsequently, the sections were incubated in normal goat serum for 1 hour, and then for 24 hours with primary antibodies against the specific axonal marker PGP 9.5 (pre-diluted mouse anti-human monoclonal antibody, ABCAM Inc, Cambridge, England; ab75447) and antibodies against the 5-HT_3A_ receptors (goat polyclonal anti-human antibody, 1:100, Santa Cruz Biotechnology Inc, Santa Cruz, CA, USA; sc-19150) and Na_V_1.8 sodium channels (rabbit anti-human polyclonal antibody, 1:200, ABCAM Inc, Cambridge, England; ab66743). The specificity of the antibodies has been previously confirmed by pre-adsorption [36,37]. Sections were rinsed with PBS and incubated with fluorescent secondary antibodies (Alexa 488 donkey-anti-goat, 1:700 for 5HT_3A_, Alexa 555 donkey-anti-mouse, 1:700 for PGP9.5, and Alexa 633 donkey-anti-rabbit, 1:700 for Na_V_1.8, Vancouver, Canada). Sections were examined with a Leica TCS SPE Confocal Microscope (Leica microsystems, Wetzlar, Germany) and images were captured for analysis using a Leica scanner attached to the microscope (Figure 1). Omission of the primary antibodies did not result in specific staining of tested sections.

#### *Analyzes of the muscle biopsies and criteria for delineation of one nerve fiber*

From each muscle biopsy a median of 4 (IQR 1) sections were analyzed.

**Figure 2:**
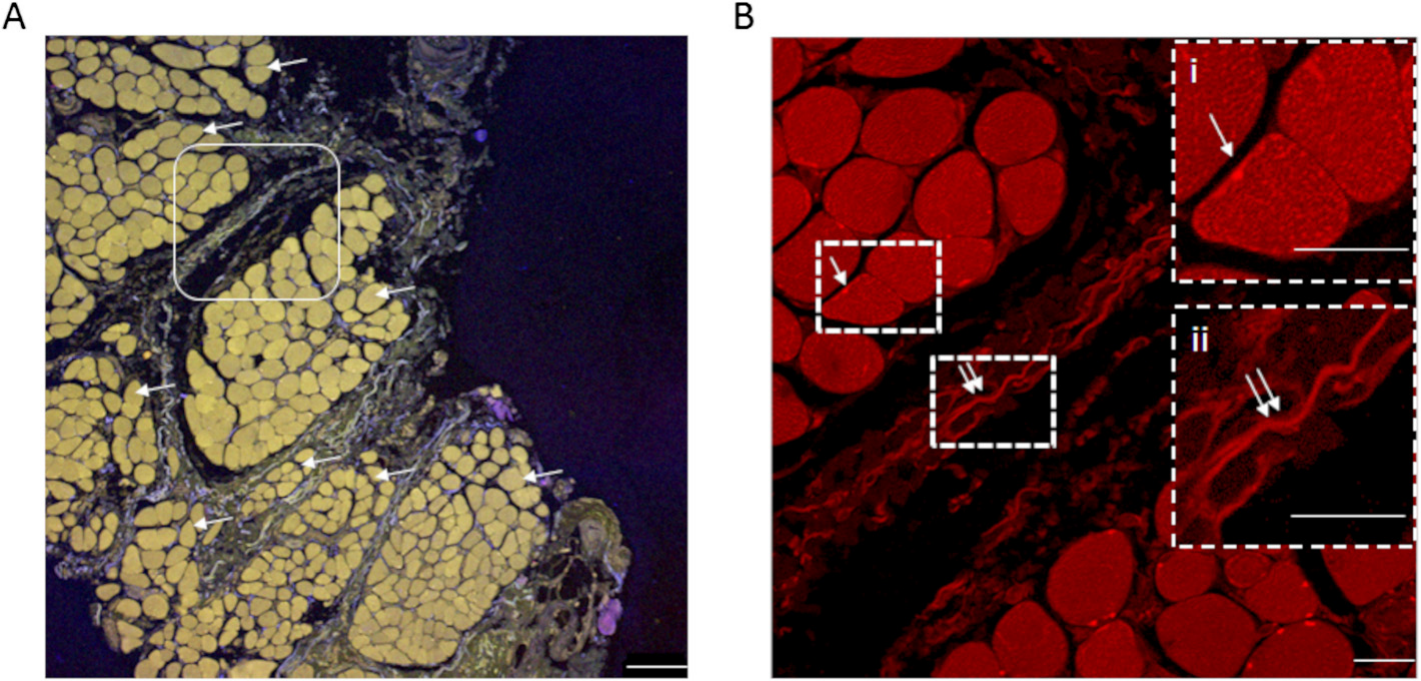
**A low power (10 x) (in A) and a high power (40 x) (in B) image of the same section of a masseter muscle (microbiopsy from a pain-free volunteer). A**. The arrows indicate individual muscle fascicles composed of myocytes separated by connective tissue. **B**. A higher power (40 x, rounded square in **A)** image of PGP 9.5 labelling with examples of fibers associated with myocytes (arrows) and connective tissue (double arrows). The insets show examples of a PGP 9.5 positive fiber associated with a myocyte (i) and with connective tissue (ii). Calibration bar **A**: 100 μm, **B**: 25 μm.

Labeling was considered positive when the intensity of the fluorescent signal exceeded the 95% confidence interval of the mean background intensity. The minimum length of a labeled fiber accepted was 1 μm.When presenting the localization of the nerve fibers they were divided into two groups, either associated with myocytes or with connective tissue. When the PGP 9.5 positive nerve fibers were in close vicinity to myocytes, i.e. long, tubular well-defined cells, they were placed in the group associated with myocytes (“arrows” in Figure 2). When the fibers were in irregular tissue surrounding the myocytes they were grouped as associated with connective tissue (“double arrows” in Figure 2).

The expression of the receptors was analyzed by assessing the amount of PGP 9.5 labeled nerve fibers that co-expressed the 5-HT_3A_ receptor alone or in combination with the Na_V_1.8 sodium channel. PGP 9.5 positive nerve fibers that co-expressed both Na_V_1.8 and 5-HT_3A_ receptors were considered as putative nociceptors. The image processing and analysis program ImageJ, (Image Processing and Analysis in Java; National Institutes of Health, USA) was used.

### Statistics

For data and statistical analyses the SigmaStat software version 3.10 (Systat software Inc., San Jose, CA, USA) was used. Data concerning the participants are expressed as mean (±SD), except for pain characteristics that are expressed with median and interquartile range (IQR) since they were either not normally distributed or assessed on an interval scale. Data regarding receptor expression was normally distributed and was not transformed. The frequencies of receptor expression are presented as mean (±SEM). To test for significant differences regarding the frequency of expression of the 5-HT_3A_ receptors and the Na_V_1.8 sodium channels as well as for significant differences between biopsy techniques Students *t*-tests were used. For all tests the level of significance was set to *P* < .05.

## Results

### Receptor expression

#### *Microbiopsies*

In microbiopsies of healthy volunteers PGP 9.5 immunoreactive fibers were identified in sections from 12 subjects (6 men and 6 women) of the masseter muscle and from 14 subjects (7 men and 7 women) of the tibialis muscle. In the masseter muscle an average of 8.2 ± 1.2 (mean ± SEM) PGP 9.5 immunoreactive fibers per section was found and in the tibialis muscle an average of 7.8 ± 1.3 fibers per section was found. Most fibers that showed PGP 9.5 immunoreactivity were associated with myocytes (masseter = 68.4%, tibialis = 87.8%).

**Figure 3:**
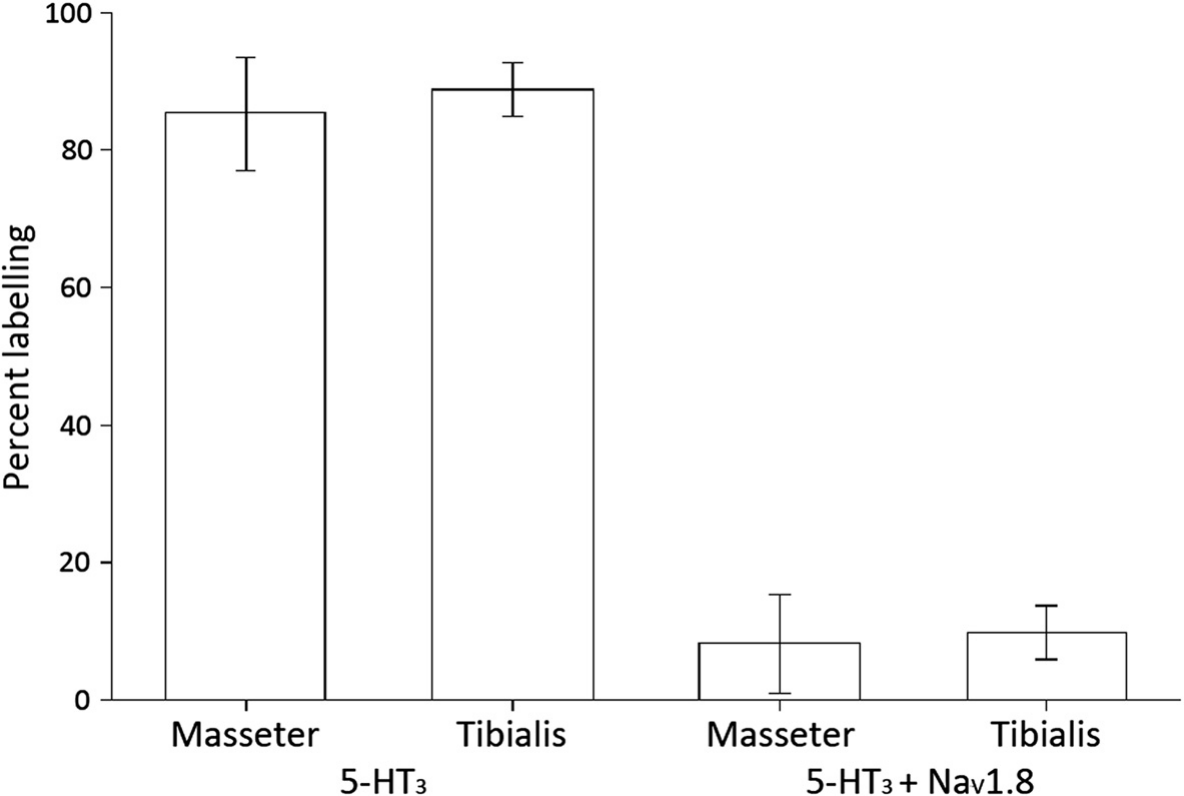
**A comparison showing the different mean frequencies for antibody labelling on PGP 9.5 immunoreactive fibers (5-HT**_**3A**_**; both 5-HT**_**3A **_**and Na**_**V**_**1.8) in the masseter and tibialis tissues.** There were no significant differences in the frequency of fibers that expressed 5-HT_3A_ or both 5-HT_3A_ and Na_V_1.8, when the two muscles were compared (*P* > .05, Students *t*-test). Each bar represents the average from all the sections obtained from each muscle type. The error bars depict standard error of the mean.

The frequency of PGP 9.5 fibers expressing the 5-HT_3A_ receptor alone or in combination with the Na_V_1.8 sodium channel are presented in Table 2, mean frequencies for antibody labelling of PGP 9.5 immunoreactive fibers are shown in Figure 3. Both in the masseter and tibialis muscles, the majority of the PGP 9.5 positive fibers expressed 5-HT_3A_ receptors. In the tibialis muscle a significantly greater number of these fibers were associated with myocytes than with connective tissue (Table 2).

**Table 2:** Presentation of the frequency (%) and location of the positive PGP 9.5 immunoreactive fibers

	**Fibers expressing 5-HT**_**3A**_			**Fibers expressing 5-HT**_**3A **_**and Na**_**V**_**1.8**		
	**Percentage of PGP 9.5 fibers**	**Location of positive fibers**		**Percentage of PGP 9.5 fibers**	**Location of positive fibers**	
		**Myocytes**	**Connective tissue**		**Myocytes**	**Connective tissue**
**Traditional biopsies**						
***Masseter muscle***						
Patients (n = 5)	**98.3%**	65.6%	34.4%	**42.6%**^**###**^	17.8%	82.2%***
Healthy (n = 5)	**96.0%**	62.5%	37.5%	**12.0%**	12.5%	87.5%***
**Microbiopsies**						
***Masseter muscle***						
Healthy (n = 12)	**85.2%**	70.5%	29.5%	**8.2%**	11.8%	88.2%***
***Tibialis muscle***						
Healthy (n = 14)	**88.7%**	86.0%	14.0%*	**9.6%**	0%	100.0%***

The table shows the frequency of the PGP 9.5 immunoreactive fibers expressing the 5-HT_3A_-receptor alone or in combination with the Na_V_1.8 sodium channel as well as the location of the positive fibers within the muscle tissue and their distribution (in association with myocytes or in the connective tissue surrounding the myocytes).

*Significantly lower frequency of positive fibers in connective tissue compared to myocytes (*P* < .011, paired *t*-test).

***Significantly higher frequency of positive fibers in connective tissue compared to in myocytes (*P* < .001, paired *t*-test).

^###^Significantly greater frequency in patients than in healthy individuals (*P* < .001, unpaired *t*-test).

In the masseter and tibialis muscles respectively, 8.2% and 9.6% of PGP 9.5 positive fibers co-expressed Na_V_1.8 and 5-HT_3A_. The vast majority of these fibers were found in connective tissue both in the masseter and in the tibialis muscles.

#### *Sex differences*

In the masseter there was no significant difference in the mean nerve fiber density when men were compared with women. There was no significant difference in the expression of 5-HT_3A_ positive fibers associated with myocytes or connective tissue between sexes (Table 3).

**Table 3:** Presentation of the mean (±SD) nerve fiber density and their distribution within the sections

	**Nerve fiber density**					
	**Fibers per section (n=)**		**Location (%) of positive fibers**			
			**Myocytes**		**Connective tissue**	
	**Men**	**Women**	**Men**	**Women**	**Men**	**Women**
**Microbiopsies**						
***Masseter muscle***						
Healthy (n = 12)	6.7 ± 1.8	5.6 ± 1.5	76 ± 15%	78 ± 20%	45 ± 20%	40 ± 24%
***Tibialis muscle***						
Healthy (n = 14)	6.7 ± 1.9	8.9 ± 1.9	95 ± 2%	77 ± 13%	26 ± 17%	38 ± 17%

The table presents the mean nerve fiber density (i.e. fibers per section), the percentage difference of positive fibers within the muscle tissue, and the distribution (either in association with myocytes or in the connective tissue surrounding the myocytes).

There are microbiopsies from the masseter muscle from six men and six women, while there are microbiopsies from seven men and seven women from the tibialis muscle.

There were no significant differences.

There was no significant difference in the mean density of fibers in the tibialis when men were compared with women. Likewise, there was no significant difference in the expression of 5-HT_3A_ positive fibers associated with myocytes or with connective tissue (Table 3).

#### *Traditional biopsies of the masseter muscle*

In the traditional masseter biopsies of healthy female volunteers (n = 5) an average of 2.7 ± 0.2 PGP 9.5 immunoreactive nerve fibers per section was found.

The vast majority of the PGP 9.5 positive fibers expressed 5-HT_3A_ receptors. Similar to what was found for the microbiopsies of the masseter muscle, the majority of nerve fibers were associated with myocytes (Table 2).

12.0% of the PGP 9.5 positive fibers were found to co-express Na_V_1.8 and 5-HT_3A_ receptors. A significantly greater number of nerve fibers that co-expressed Na_V_1.8 and 5-HT_3A_ was associated with connective tissue than with myocytes (Table 2).

In the masseter muscle of female patients (n = 5) an average of 3.8 ± 3 PGP 9.5 positive fibers per section was found. Virtually all PGP 9.5 positive fibers expressed 5-HT_3A_ receptors. Similar to healthy individuals, the majority of these nerve fibers were associated with myocytes.

42.6% of the PGP 9.5 positive fibers in the masseter of the patients co-expressed Na_V_1.8 and 5-HT_3A_ receptors. There was a significantly higher co-expression of Na_V_1.8 and 5-HT_3A_ positive fibers that were associated with connective tissue than with myocytes (Table 2).

#### *Differences between TMD patients and pain-free controls*

The total number of PGP 9.5 positive fibers per section that expressed 5-HT_3A_ receptors was significantly higher in female patients (3.79 ± 0.75) compared to healthy females (2.37 ± 0.58) (*P* = .010). Also the frequency of PGP 9.5 positive fibers that co-expressed Na_V_1.8 and 5-HT_3A_ receptors was significantly higher than that found in healthy women (Table 2; and as an example see Figure 1).

#### *Differences between the biopsy techniques*

Both techniques provided an adequate muscle section. However, the muscle sections obtained with the microbiopsy technique were significantly smaller in volume and in weight as well as in the area of the wound at the entrance to the muscle. Further, the microbiopsies caused less discomfort and fewer visits for the individual undergoing the procedure (Table 4).

**Table 4:** Comparison of the two techniques used for obtaining masseter biopsies from healthy volunteers

	**Microbiopsies (n = 18)**	**Traditional biopsies (n = 10)**
**Mean weight of each muscle section**	19.8 (±2.5) mg***	58.4 (±9.6) mg
**Mean volume of each muscle section**	13.0 (±1.6) mm^3^***	64.4 (±9.9) mm^3^
**Surrounding tissues**	No	Yes
Type of tissue		Parotid gland (20%)
		Fat cells (30%)
**Adequate muscle section**	100%	100%
**Number of PGP 9.5 positive fibers per section**	8.2 ± 1.2*	2.7 ± 0.2
**Area of wound**	1 (±0.04) mm^2^***	10 (±0.7) mm^2^
**Post-surgical discomfort**	3 (±1) days	8 (±2) days
Type of discomfort		
	Palpatory tenderness over the belly of the masseter (100%)	Palpatory tenderness over the belly of the masseter muscle (100%)
	Bleeding (6%)	Limited jaw function (10%)
		Chewing difficulties (50%)
		Discomfort from sutures (100%)
		Extra visit to remove sutures (100%)

Differences are presented as mean (±SD) values.

*P < .05, unpaired *t*-test, ***P < .001, unpaired *t*-test.

The number of PGP 9.5 positive fibers per section was significantly higher in the microbiopsies of healthy women than in traditional biopsies from the healthy women (Table 4), but there were no significant differences in the receptor expression between the two biopsy techniques. In the microbiopsy group, 70.5% of the fibers associated with myocytes expressed 5-HT_3A_ receptors while the corresponding number was 62.5% in the traditional biopsy group. Further 29.5% of the fibers associated with connective tissue fibers expressed 5-HT_3A_ receptors in the microbiopsy group, whereas the corresponding number was 37.5% in the traditional biopsy group (Table 2).

## Discussion

### Main findings

The main findings of this study were that the 5-HT_3A_ receptor is highly expressed in human masseter and tibialis muscle tissue, but that there were no differences in expression due to sex. Further, there were more immunoreactive nerve fibers expressing 5-HT_3_ receptors and Na_V_1.8 sodium channels in the masseter muscle of female patients with myofascial TMD compared to healthy female controls. These findings indicate that 5-HT_3_ receptors might be up-regulated in myofascial TMD and may therefore play a role in pain transmission, hence providing the 5-HT_3_-receptor as a target for future therapeutics or as a potential biomarker of chronic muscle pain.

Secondarily, the results from this study indicate that the microbiopsy technique provided sufficient muscle tissue, less discomfort and post-surgical pain than the traditional biopsy technique. Therefore, the microbiopsy technique can be regarded as a valid biopsy method for immunohistochemical analyses of muscle tissue, and may, thus, be preferred to traditional open biopsies in future studies or even as a diagnostic tool.

### Receptor expression

The immunohistochemical analysis of the muscle sections indicates that the 5-HT_3A_ receptor is highly expressed by human masseter and tibialis anterior muscle nerve fibers. To our knowledge there are no other studies that have studied the expression of the 5-HT3 receptor in human muscle tissues. A recent study did show that the NMDA receptor is also expressed by masseter muscle nerve fibers (45-50%) in humans and rats [22]. However, results from animal studies regarding the expression of the 5-HT_3_ receptor in trigeminal and dorsal root ganglion neurons [7,38] are consistent with the present study. It is well established that the Na_V_1.8 sodium channels play an important role in nociception and chronic pain when found on small unmyelinated and thinly myelinated sensory neurons [24,25,29,30,39] since they contribute to action potentials in sensory neurons [28,39,40] and are involved in peripheral sensitization [23]. They have further also been found in large diameter sensory neurons in human pain states [41], as well as large diameter fibers in mouse [42] and rat [26,43]muscle afferent neurons. Hence, this TTX resistant sodium channel is not just limited to small diameter nerve fibers [44]. Inflammation has been shown to enhance nociceptive signaling by the Na_V_1.8 sodium channels [31,44-46], in part, through release of inflammatory mediators, such as 5-HT and prostaglandin E_2_, which directly increase Na_V_1.8 mediated currents [31,47]. Also, 5-HT is known to participate in pain mediation at the peripheral level, acting on the 5-HT_3_ receptors, which mainly appear on afferent nociceptive sensory, autonomic and enteric neurons [16]. Given this, one can assume that the Na_V_1.8 positive fibers are sensory neurons with a potential nociceptive function, consequently implying that the muscle nerve fibers that co-express the 5-HT_3A_ receptors and Na_V_1.8 sodium channels are most probably nociceptors.

Noteworthy is the significantly higher average of fibers per section in the masseter muscle of patients with myofascial TMD, which could indicate a proliferation of nerve fibers in the painful muscle tissue. There is some evidence that hyper-innervation of the tendon with putative nociceptive fibers occurs in Achilles tendinosis [48,49]. The larger number of fibers in tender regions of the masseter muscle of TMD patients found in this study could indicate an up-regulation of 5-HT_3_ receptors. This finding may explain previous findings that a local injection of the 5-HT_3_ receptor antagonist granisetron was effective in decreasing muscle sensitivity in patients with localized myalgia [21] and fibromyalgia [50].

The majority of the 5-HT_3A_ and Na_V_1.8 positive fibers in the masseter muscle were found in the connective tissue, which suggests that masticatory muscle pain may emanate primarily from the connective tissue. This theory is based on the findings from the muscle sections where both the patients and healthy subjects had significantly more Na_V_1.8 positive nerve fibers in connective tissue compared to fibers associated with myocytes. The elevated levels of serotonin in the masseter muscle in patients with myofascial TMD [3] and the very high expression of 5-HT_3A_ and Na_V_1.8 positive fibers on sensory nerves associated with connective tissue, shown in this study, support the theory that the muscle nociceptors in painful muscles could be sensitized by serotonin and hence that serotonin may have an important role in myofascial TMD.

Apart from pain mediation, the 5-HT_3_ receptors are involved in many events in the human body. Centrally they play an important role in psychosis, anxiety, cognition and eating disorders [51], but also in motor system function. A recent study showed that activation of the 5-HT_3_ receptor induced rhythmic movements of the hindlimbs in mice [52]. In the periphery they have a well-known role in emetic pathways [53] and they have a role in the intestinal tone, activating colonic migrating motor complex, i.e. muscular motor activity [54]. PGP 9.5 labels both small and large diameter myelinated fibers [55] and Na_V_1.8 is a marker of small diameter thinly myelinated nociceptive fibers [44]. With this in mind, as the majority of the 5-HT_3A_ positive muscle nerve fibers were located near myocytes and did not co-express Na_V_1.8, our findings imply that the 5-HT_3A_ positive fibers in association with the myocytes are likely either proprioceptive muscle spindle afferent fibers or motor axons [56].

### Comparison of the biopsy techniques

The results of this study indicate that the microbiopsy technique provided sufficient muscle tissue for immunohistochemistry and led to less discomfort and post-surgical pain as well as fewer visits than the traditional biopsy technique. This is in agreement with a previous study comparing the microbiopsy technique with the traditional open biopsy technique in the vastus lateralis of the quadriceps femoris muscle [57]. Further, the microbiopsy technique seems to be more precise, since by using the Bard®TruGuide™ coaxial needle, the surgeon is more likely to remove tissue from the exact region of interest without damaging the surrounding tissue [35]. It was also shown that the muscle sections from the microbiopsies contained less of the surrounding tissues, such as parts of the salivary glands than traditional open biopsies (Table 4). Taken together this shows that the microbiopsy technique is a valid biopsy method for immunohistochemical analyses of muscle tissue and hence, may be preferred to traditional open biopsies in future studies or even as a diagnostic tool.

### Differences due to sex or anatomical localization

In contrast to a previous study [7] indicating that the expression of 5-HT_3_ receptors by trigeminal ganglion neurons innervating the masseter muscle was higher in female rats, this study showed no significant difference in the expression of 5-HT_3A_ receptors in the masseter or tibialis muscles, when men and women were compared.

In previous studies, women reported more pain than men when serotonin was injected into the masseter muscle [13], and a larger pain area when hypertonic saline was injected [10]. Based on those studies one could have expected a significant sex difference with a larger amount of nerve fibers co-expressing 5-HT_3A_ receptors and Na_V_1.8 sodium channels in women. Further, a previous human experimental study showed that systemic administration of the 5-HT_3_ receptor antagonist granisetron increased the PPT over the tibialis anterior muscle in men but not in women [11]. As a possible explanation for this difference could have been that the number of receptors differs between men and women, however, the findings of the present study do not support this explanation. It should be noted that a limitation of this study is the small sample size (6–7 individuals of each sex), which may have been insufficient to permit identification of sex-related differences in 5-HT_3A_ receptor expression.

There was a difference between the two skeletal muscles since a majority of the fibers co-expressing 5-HT_3A_ receptors and Na_V_1.8 sodium channels were found in the connective tissue in the masseter muscle while in the tibialis muscle these fibers were found to be associated with myocytes. The tibialis muscle is rarely exposed to chronic pain in contrast to the masseter muscle and is further innervated by the deep peroneal nerve which major role is to contract the muscle fibers in order to dorsiflex and invert the foot [58]. In contrast to the tibialis anterior muscle, the masseter muscle expressed a larger amount of putatively nociceptive nerve fibers in the connective tissue. This is an interesting difference between muscles that could be a consequence of the different innervation with either trigeminal branches (masseter muscle) or spinal branches (tibialis muscle) and functionality of the nerves regarding motor and sensory functions [58]. Therefore, the larger amount of putatively nociceptive nerve fibers in the connective tissue of the masseter muscle and the difference in innervation might partly explain the more frequent presence of chronic myalgia in the masseter muscle.

### Study limitations

One limitation of the study is, as mentioned, the small sample size which might have affected the outcome regarding sex-related differences. Another possible limitation of the study is the exclusion criterion “use of analgesic or anti-inflammatory medication during the 24 hours preceding the biopsy”. This criterion was primarily in order to avoid any risk of including a patient with myofascial TMD among the healthy participants. However, 24 hours is a short wash-out period for NSAIDs which could have affected the biopsy procedure negatively, for example through increased bleeding, since the use of NSAIDs might impair the thrombocyte aggregation for up to 8–10 days. Use of NSAID might also have suppressed receptor expression. If so, it is likely that patients with myfascial TMD would have been affected more and in that case the difference in frequency would be even greater than found in the current study. The storage of the biopsies in paraffin could be another possible limitation since this technique might blunt the antigenicity of the samples, which may make it more difficult to detect the receptors. In future studies the muscle biopsies are suggested to be frozen immediately in -80°C.

## Conclusions

This study showed that the 5-HT_3A_ receptor is highly expressed by human masseter and tibialis muscle nerve fibers. Further, a greater number of putative nociceptors were found to express the 5-HT_3_ in the masseter muscle of female patients with myofascial TMD compared to healthy female controls, which suggests that 5-HT_3_ receptors might be up-regulated in myofascial TMD. This finding could support the use of 5-HT_3_ receptor expression in muscle biopsies as a biomarker of chronic masticatory muscle pain.

## Abbreviations

TMD: Temporomandibular disorders; 5-HT: 5-hydroxytryptamine serotonin; TTX: Tetrodotoxin; Na_V_s: Sodium channel; RDC/TMD: Research diagnostic criteria for temporomandibular disorders; PBS: Phosphate-buffered saline.

## Competing interests

The authors declare that they have no competing interests.

## Authors’ contributions

NC, contributed in the design of the study, by collecting the material (microbiopsies), in the statistical analysis, and as the main author of the manuscript. IK contributed in the analysis of the material and in the result section of the manuscript. BEC, contributed in the design of the study, in the analysis of the material, in the statistical analysis, and in the manuscript in general. UK, contributed in the analysis of the material and in the discussion section of the manuscript. XD, contributed in the analysis of the material and in the discussion section of the manuscript. AR, contributed by collecting the material (traditional biopsies) and in the discussion section of the manuscript. SK, contributed in the design of the study and in the result and discussion section of the manuscript. ME, contributed in the design of the study, in the statistical analysis, and in the manuscript in general. All authors read and approved the final manuscript.
